# Heterologous and cross-species tropism of cancer-derived extracellular vesicles

**DOI:** 10.7150/thno.34824

**Published:** 2019-08-09

**Authors:** Mariangela Garofalo, Alessandro Villa, Daniela Crescenti, Monica Marzagalli, Lukasz Kuryk, Patrizia Limonta, Vincenzo Mazzaferro, Paolo Ciana

**Affiliations:** 1 Department of Oncology and Hemato-Oncology, Center of Excellence on Neurodegenerative Diseases, University of Milan, Milan, Italy; 2 Department of Pharmacological and Biomolecular Sciences, University of Milan, Milan, Italy; 3 Targovax Oy, Clinical Science, Helsinki, Finland; 4 National Institute of Public Health - National Institute of Hygiene, Department of Virology, Warsaw, Poland; 5 Istituto Nazionale Tumori Fondazione IRCCS, Milan, Italy

**Keywords:** extracellular vesicles, oncolytic adenoviruses, cancer therapy, immunocompetent cancer mouse models, *in vivo* imaging.

## Abstract

Extracellular vesicles (EVs) are naturally occurring cargo delivery vesicles that have recently received considerable attention for their roles in intercellular communication in many physiological and pathological processes, including tumourigenesis. EVs generated by different tissues demonstrated specific homing: in particular, cancer-derived EVs showed a selective tropism for the tumor tissue from which the vesicles originated. For this property, EVs have been proposed as drug delivery tools for anti-cancer therapies, although the limited knowledge about their *in vivo* tropism hinders their therapeutic applications. The current study aimed to characterize the targeting properties of cancer-derived EVs *in vitro* and their biodistribution *in vivo*, by using an imaging approach.

**Methods**: EVs were generated from: i) murine lung (LL/2) and colon (MC-38) cancer lines, ii) human lung cancer cell line (A549) and iii) human liver biopsy samples from healthy individuals. EVs were loaded with fluorescent dyes alone or in combination with a biopharmaceutical agent, the oncolytic adenovirus (OV), characterized for charge and size and tested for their activity in cancer cell lines. Finally, optical imaging was extensively applied to study *in vivo* and *ex vivo* the biodistribution of EVs originated from different sources in different mouse models of cancer, including xenograft, syngeneic graft and the MMTV-NeuT genetically modified animal.

**Results**: We initially demonstrated that even loading EVs even with a large biopharmaceutical oncolytic viruses (OVs) did not significantly change their charge and dimension properties, while increasing their anti-neoplastic activity compared to the virus or EVs alone. Interestingly, this activity was observed even if the EVs derived from lung cancer were applied to colon carcinoma cell lines and *vice versa*, suggesting that the EV uptake occurred *in vitro* without any specificity for the cancer cells from which the vesicles originated. When administered i.v (intravenously) to the mouse models of cancer, the tumour-derived EVs, but not the EVs derived from a healthy tissue, demonstrated a selective accumulation of the fluorescence at the tumour site 24 h after injection; adding OVs to the formulation did not change the tumour-specific tropism of the EVs also *in vivo*. Most interestingly, the *in vivo* experiments confirmed the *in vitro* observation of the generalized tropism of tumour-derived EVs for any neoplastic tissue, independent of the tumour type or even the species originating the vesicles.

**Conclusions**: Taken together, our *in vitro* and *in vivo* data demonstrate for the first time a heterologous, cross-species tumour-tropism for cancer-derived EVs. This finding challenges our current view on the homing properties of EVs and opens new avenues for the selective delivery of diagnostic/therapeutic agents to solid tumours.

## Introduction

Intercellular communication regulates both biological and pathological processes 1.

Cells continuously cross-talk by using different networks such as paracrine signalling, transport through gap junctions and extracellular vesicles (EVs) 1. EVs are a heterogeneous group of cells-derived membrane vesicles that are present in body fluids and are able to carry proteins, lipids and coding or noncoding RNA molecules 2. These vesicles participate in the intercellular communication under both physiological and pathological conditions; in healthy individuals they are involved in the regulation and maintenance of reproduction, cell death, tissue repair and inflammation 3, while in tumourigenesis, EVs contribute to the acquisition of different hallmarks of cancer including inhibition of cell death, invasion, metastasis and immunosuppression 4,5. In this scenario EVs are emerging as key mediators in pathological processes and can act at local or systemic levels 6. The mechanism by which the vesicles actively participate in the neoplastic transformation may involve the transfer of proteins, lipids and nucleic acids after a physical interaction with target cells 7,8.

Several mechanisms have been proposed for EV uptake 9: EVs can either fuse with the cellular plasma membrane to deliver their cargo 10, or be internalized by cells. It has also been suggested that EVs could use the energy-dependent receptor-mediated endocytosis, including clathrin-mediated endocytosis, caveolin-mediated endocytosis 11,12, micropinocytosis 13 and phagocytosis 14. Since the EV internalization process could occur through multiple pathways, the exact mechanisms driving the EVs' delivery to a specific target cell remain elusive, although selected protein-protein interactions have been proposed for some tissue-specific uptake 12,15 as in the case of tumour derived EVs that preferentially home to the cancer tissue originating the vesicles compared to the healthy tissues 15. This tumor tropism may be the consequence of functional properties specifically acquired by the cancer-derived EVs, which have different biogenesis mechanisms 16,17, cargo uptake 18,19 and expression of specific membrane proteins and antigens 20,21, compared with vesicles originating from non-malignant cells.

EVs'tumor-specific tropism, absence of immunogenicity, natural composition, and ability to be loaded with small molecules and biologics are highly attractive features that raise the possibility of using EVs as carriers for theranostics 22-24. Indeed, EVs were recently used for the delivery of OVs 25-27 and chemotherapeutic drugs 28, providing evidence of a tumour-targeted delivery and increased efficacy of the treatments in preclinical models 24. Although highly attractive, the use of EVs in clinical applications remains limited because the molecular basis of their tropism and targeted delivery to cancer cells has not been thoroughly elucidated to date, which is the basis for developing an effective EV-driven anti-neoplastic therapy 29. Indeed, it is still not clear whether the tumour tissue from which the EVs originate dictates their strict tissue tropism 15,30,31, since there are controversial reports indicating that this tumour specificity may be lacking 9. Thus, despite the intense research in the field, little is known about the EVs' biodistribution and their *in vivo* trafficking 32-34.

In this study, we investigated the tropism of murine lung and colon cancer-derived EVs labelled with fluorescent dyes 24,35 *in vitro* and *in vivo*, by using imaging technology. We demonstrated the existence of a heterologous, cross-species cancer-specific homing of the vesicles. This homing capability is not modified by loading the vesicles with a large therapeutic agent such as an OV 36-40, suggesting the possibility of generating EVs from cell lines even from different species which can be used to deliver theranostics to different tumour types.

## Materials and Methods

### Cell culture

LL/2 mouse lung cancer cell line, MC-38 mouse colon cancer cell line, C2C12 mouse myoblast cell line and A549 human lung cancer cell line were purchased from the American Type Culture Collection (ATCC, USA). The cells were cultured at 37 °C and 5 % CO_2_ in Dulbecco's modified eagle medium (DMEM, Lonza, Switzerland) supplemented with 10 % fetal bovine serum (FBS, Gibco Laboratories, USA), 1 % of 100 u/mL penicillin/streptomycin (Gibco Laboratories) and 1% L-glutamine (Gibco Laboratories).

### Oncolytic virus

Oncolytic adenovirus Ad5/3-CD40L has been characterized by molecular analyses (PCR, sequencing) and functionality assay (CD40 ligand), 41 to check virus genome stability, integrity and oncolytic properties. Viral stocks were expanded in human lung cancer cell line A549 and purified on cesium chloride gradients. The viral particle concentration was determined by OD_260_-reading (VP/mL) and standard TCID_50_ (tissue culture infectious dose 50) assay was performed to determine infectious particle titer.

### Production of EVs, Lipophilic Dye loaded EVs and Indocyanine green loaded EVs

In order to produce EVs 2.6 ×10^6^ LL/2, 5 ×10^6^ MC-38 and 2.6 x 10^6^ A549 cells were plated into T-175 flask in medium supplemented with 5 % FBS. The FBS growth media was ultra-centrifuged overnight (110 000 x g at 4°C for 18 h, Optima LE-80K ultracentrifuge, rotor type 50.2, Beckman Coulter) to remove EVs present in the serum.

EVs were isolated from the conditioned medium using differential centrifugation steps. First the conditioned medium was centrifuged at 500 x g in 4°C for 10 min to pellet cells (Allegra X-15R Centrifuge, Beckman Coulter). Then, the supernatant was collected and ultra-centrifuged for 2 h at 100 000 x g in 4°C, using Optima L-80 XP ultra-centrifuge (Beckman Coulter) with rotor SW32Ti (Beckman Coulter). The supernatant was aspirated and EV- containing pellets re-suspended in PBS (Lonza) 100 μL and stored for few days at - 80 °C.

EVs from patient-derived liver tissue were isolated using different ultracentrifugation steps. Firstly, liver tissue was minced and passed through a 0.2 micron filter for subsequent ultra-centrifugation. Then, samples were ultra-centrifuged for 2 h at 100 000 x g and 4°C, using Optima L-80 XP ultra-centrifuge (Beckman Coulter) with rotor SW32Ti (Beckman Coulter). The supernatant was aspirated and pellets containing EV-Virus re-suspended in PBS 100μL and stored for few days at - 80 °C.

EVs from LL/2 and MC-38 cells were loaded with DiD lipophilic dye (EV-DiD) as previously described 24,42 and prepared by incubating 1 × 10^8^- 5 ×10^9^ EVs for 1 hour at RT with 5 μL of DiD (Biotium) per mL of EV suspension in PBS. Next, the samples were centrifuged at 150 000 x g for 3 h to pellet the EVs. The supernatant containing unbound DiD was removed, and the EV-pellet was washed by suspending it in PBS and pelleting it again at 150 000 x g.

EVs from LL/2 and MC-38 cells were loaded with Indocyanine green (EV-ICG) and prepared by incubating 1× 10^8^-5×10^9^ EVs in PBS for 12 h at 4°C with 10ug/mL ICG (Sigma). Next, the samples were centrifuged at 150 000 x g for 3 h to pellet the EVs. The supernatant containing unbound ICG was removed, and the EV-pellet was washed by suspending it in PBS and pelleting it again at 150 000 x g.

### Production of EV-Virus formulations

EV-encapsulated virus (EV-Virus) were produced as previously described 23,24,42, 2.6 x 10^6^ of LL/2 cells and 5×10^6^ of MC-38 were infected with 10 viral particles/cell of Ad5D24 and were cultured at 37 °C and 5 % CO_2_. 48 h later when most of the cells were detached from the culture flask, the culture media were collected for EV-Virus isolation using differential centrifugation. First the conditioned medium was centrifuged at 500 x g and 4°C for 10 min, to separate the cells (Allegra X-15R Centrifuge, Beckman Coulter). Then, the supernatant containing EV-Virus was collected and ultra-centrifuged for 2 h at 100 000 x g and 4°C, using Optima L-80 XP ultra-centrifuge (Beckman Coulter) with rotor SW32Ti (Beckman Coulter). The supernatant was aspirated and pellets containing EV-Virus re-suspended in PBS 100μL and stored at - 80 °C. EV-Virus samples were incubated in 100 mM NaOH at room temperature for 20 min in order to inactivate any free not EV encapsulated virus present. Free virus used as controls was always inactivated for each experiment performed as previously reported 23,43. Samples were subsequently neutralized by the addition of HCl 0.1 M.

To generate EV-DiD-Virus, the EV-Virus formulation was incubated for 1 h at RT in 5 μL of DiD per mL of EV suspension in DPBS. Samples were then centrifuged at 150 000 x g for 3 h at RT, in order to pellet EV-DiD-Virus. The washing procedure was repeated using PBS as diluent. The final EV-DiD-Virus pellet was re-suspended in 100 μL of PBS and stored for few days at -80 °C until use.

### Size distribution analysis by nanoparticle tracking analysis (NTA)

Size distribution and concentration of EV-LL/2, EV-MC-38, Virus, EV-Virus-LL/2 and EV-Virus-MC-38 formulations were analyzed by NTA using Nanosight model LM14 (Nanosight) equipped with blue (404 nm, 70 mV) laser and sCMOS camera. The samples containing virus were incubated at +95 °C for 10 min in order to inactivate the viruses. NTA was performed for each sample by recording three 90 s videos, subsequently analyzed using NTA software 3.0 (Nanosight). The detection threshold was set to level 5 and camera level to 15.

### Zeta potential analysis by electrophoretic light scattering

The zeta potential was measured using ZetaSizer Nano (Malvern, UK). All the samples were diluted in a volume of 800 μL of MilliQ H_2_O and injected with a 1 mL syringe in the capillary flow (DTS1070 folded capillary cell) for the measurement. An equilibration time of 120 s was set on the software to allow the samples to stabilize at 25°C inside the measurement chamber. Three parallel measurements were performed on each sample.

### Cryo-EM

Cryo-EM images were acquired with a FEI Talos Arctica 200 kV FEG electron microscope equipped with a FEI Falcon 3EC direct electron detector and Volta Phase-plate. Prior to Cryo-EV imaging, samples were vitrified on a FEI Vitrobot IV apparatus, and processed as previously reported.

### Western blot analysis

Extracellular vesicles preparation (equivalent to 60 μg of protein lysates) were boiled at 95^o^C for 5 min, separated on 4-10% SDS-PAGE using beta-mercaptoethanol as reducing agent and transferred into nitrocellulose membranes (Amersham). Membranes were then blocked in 5% non-fat dry milk in TBS-T (0.2% Tween-20) at RT and incubated overnight with the primary antibodies against the exosomal marker CD63 (SAB4301607 Sigma, 1:1000), TSG101 (4A10 Abcam, 1:500) and CD9 (C9993 Sigma, 1:500). Immuno-reactive bands were visualized with chemiluminescence by using ECL^TM^ Western Blotting Analysis System according to the manufacturer's instructions (Amersham).

### MTS cell viability assay

LL/2 and MC-38 cells were seeded at a density of 1×10^4^ cells/well in 96-well plates and maintained under standard growth condition. On the following day LL/2 cells were treated in triplicates with EVs isolated from LL/2 cells (EV-LL/2) (10 particles/cell), EVs isolated from MC-38 cells (EV-MC-38) (10 particles/cell), Virus (10vp/cell), EV-Virus from LL/2 cells (EV-Virus-LL/2) (10 particles/cell), EV-Virus from MC-38 cells (EV-Virus-MC-38) (10 particles/cell). MC-38 cells were treated in triplicates with EVs isolated from MC-38 cells (EV-MC-38) (10 particles/cell), EVs isolated from LL/2 cells (EV-LL/2) (10 particles/cell), Virus (10vp/cell), EV-Virus from MC-38 cells (EV-Virus-MC-38) (10 particles/cell), EV-Virus from LL/2 cells (EV-Virus-LL/2) (10 particles/cell). Cell viability was determined by MTS assay according to the manufacturer's protocol (Cell Titer 96 AQueous One Solution Cell Proliferation Assay; Promega, Nacka, Sweden). The absorbance was measured with a 96-wells plate spectrophotometer Varioskan Flash Multimode Reader (Thermo Scientific) at 490 nm. The experiments were independently performed three times with triplicates of each condition in each experiment.

### Immunogenicity of tumor cell death* in vitro*

Calreticulin (CRT) exposure. LL/2 and MC-38 cells were seeded in duplicate onto 6 well plates at 5×10^5^ cells/well. LL/2 cells were treated in triplicates with EV-LL/2 (10 particles/cell), EV-MC-38 (10 particles/cell), Virus (10vp/cell), EV-Virus-LL/2 (10 particles/cell), EV-Virus-MC-38 (10 particles/cell). MC-38 cells were treated in triplicates with EV-MC-38 (10 particles/cell), EV-LL/2 (10 particles/cell), Virus (10vp/cell), EV-Virus-MC-38 (10 particles/cell), EV-Virus-LL/2 (10 particles/cell). After 24 h cells were harvested and stained with 1:1000 diluted rabbit polyclonal anti-Calreticulin antibody (Abcam, Cambridge, UK) for 40 min at 4°C subsequently with 1:100 diluted Alexa-Fluor 488 secondary antibody (Invitrogen, Carlsbad, CA) and analyzed by flow cytometry (LSR II, BD, Franklin Lakes, NJ).

ATP release. LL/2 and MC-38 cell lines were seeded in triplicates onto 96 well plates at 1×10^4^ cells/well and treated as mentioned above. Supernatants were collected after 48 h and analyzed with ATP Determination Kit according to manufacturer's protocol (Promega, Madison, WI) for luminometric analysis (Varioscan Flash, ThermoFisher Scientific, Waltham, MA).

### *In vivo* biodistribution study

All the animal experiments were performed and approved by the Italian Ministry of Research and University (permission numbers: 12-12-30012012, 547/2015) and controlled by a Departmental panel of experts. C57BL/6 and MMTV-NeuT mice were used for the experiments. The acclimatization period was 14 days prior to LL/2 and MC-38 cancer cell injections. Health status of the mice was monitored daily and as soon as signs of pain or distress were evident they were euthanized. Administration of any EV formulation did not produce sign of pain or changes in the behavior in the treated animals. Murine xenografts were established by injecting 2×10^6^ LL/2 and 1×10^6^ MC-38 cells s.c. into the neck of 12-week old male mice. The aim of the fluorescence study was to have a direct evidence of the specific tropism of different cancer derived EVs encapsulated with oncolytic viruses to the tumor, thus the fluorescent emission of a lipophilic dye (DiD) and Indocyanine green (ICG) was related to the particle biodistribution. Therefore, we performed the following treatments: DiD (5 μL per mL of EV suspension in DPBS), EV-DiD-LL/2 (n=5) (1×10^8^ particles/tumor), EV-DiD-MC-38 (n=5) (1×10^8^ particles/tumor), EV-DiD-Virus-LL/2 (n=5) (1×10^8^ particles/tumor + 1×10^8^ vp/tumor), EV-DiD-Virus-MC-38 (n=5) (1×10^8^ particles/tumor + 1×10^8^ vp/tumor), ICG (10 ug/mL), EV-ICG-LL/2 (1×10^8^ particles/tumor + 10 ug/mL). Treatment groups were administered i.v (50 µl) to mice with tumors (one tumor per mouse about 5 mm in diameter). Brain, tumors, livers and serum from C57BL/6 mouse were collected for quantitative real time PCR (LighCycler 480, Roche, Basel, Switzerland).

### Patient samples

Human liver tissues were obtained during planned surgical interventation for benign liver diseases (mainly cholecystectomy for stones or chronic cholecistis) performed by the Hepato-Pancreato-Biliary Unit at the Istituto Nazionale Tumori, Milan. The use of human materials was conducted in accordance with the declaration of Helsinki.

### *In vivo* and *ex vivo* imaging

The *in vivo* fluorescence imaging was carried out 24 h post i.v. EV treatments, mice were anaesthetized using Isofluorane (Isofluorane-Vet; Merial, Lyon, France) and kept under anesthesia during imaging sessions carried out with the Imaging System (5 min for dorsal view and 5 min for ventral view) (IVIS Lumina II Quantitative Fluorescent Imaging; PerkinElmer, Waltham, MA, USA) with suitable filters (Cy5.5, ICG) and following the manufacturer instructions for fluorescence background subtraction. For the *ex vivo* imaging acquisition of fluorescence mice were euthanized by cervical dislocation and *ex vivo* imaging of the selected organs was carried out immediately after death over an exposition time of 1 second. The quantification was done with Living Image Software 3.2 (PerkinElmer).

### Quantitative real-time PCR

qPCR for adenovirus E4 copy number was carried out according to the protocol previously described 44 primer FW: 5'-GGA GTG CGC CGA GAC AAC-3', primer RV: 5'-ACT ACG TCC GGC GTT CCA T-3', probe E4: 5'-(6FAM)-TGG CAT GAC ACT ACG ACC AAC ACG ATC T- (TAMRA)-3'^45^. Total DNA was extracted from LL/2 cells 48 h post treatment *in vitro* and from resected brains, tumors, livers, blood from C57BL/6 mouse model after 24h post treatment, using the QIAamp DNA Blood Mini Kit (Qiagen, Hilden, Germany) according to manufacturer's protocol. Subsequently isolated DNA was analyzed for adenoviral E4 copy number normalized to murine beta-actin (liver, blood) ((primer FW: 5'-CGA GCG GTT CCG ATG C-3', primer RV: 5'-TGG ATG CCA CAG GAT TCC AT-3', probe murine beta-actin: 5'-(6FAM)-AGG CTC TTT TCC AGC CTT CCT TCT TGG-(TAMRA)-3'. Samples were analyzed using LighCycler qPCR machine (LighCycler 480, Roche, Basel, Switzerland).

### Quantitative assay for detection of hCD40L

Quantitative analysis of human CD40L (hCD40L) produced by the investigated oncolytic adenovirus was performed using Elisa kit (Abcam, ab99991) as previously described 41. Standards and samples were pipetted into the wells to the immobilized antibody. The wells were washed and a biotinylated anti-hCD40L antibody was added. After washing away the unbound biotinylated antibody, horse radish peroxidase (HRP)-conjugated streptavidin was pipetted to the wells. The wells were again washed, a TMB substrate solution was added to the wells, and color development was monitored at 450 nm, which was proportional to the amount of bound CD40L. The mean absorbance for each set of standards and samples were calculated after subtracting the absorbance of the negative control. The standard curve was plotted immediately after stopping the reaction, with standard concentration on the y-axis and absorbance on the x-axis, and the best-fit straight line was drawn through the standard points.

### Statistical analysis

Statistical significance was analyzed by using one-way ANOVA with Tukey's Multiple Comparison test and nonparametric Mann-Whitney test. All statistical analysis, calculations and tests were performed using GraphPad Prism 5 (GraphPad Software, San Diego, CA).

## Results

### Production of LL/2 and MC-38 derived EV formulations and their ability to induce immunogenic cell death

**Figure 1:**
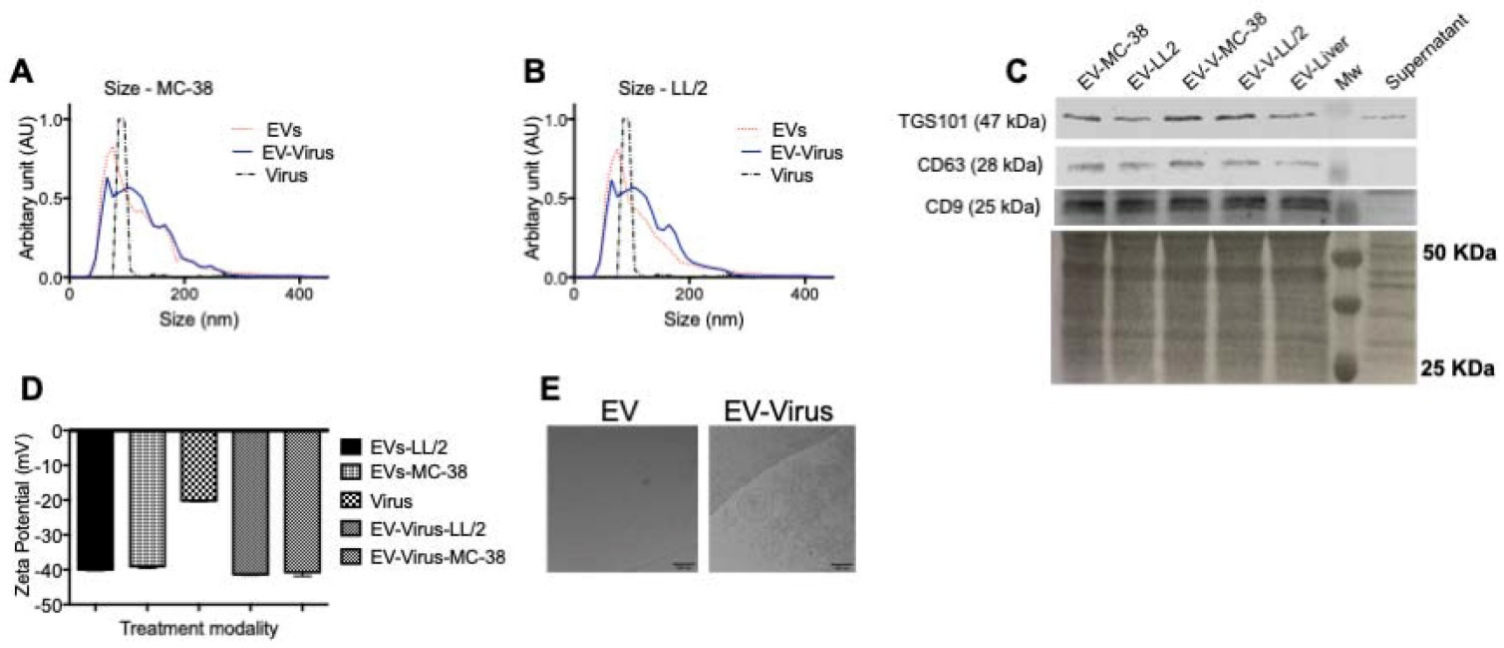
** Size and net-charge of murine lung and colon cancer derived extracellular vesicles**. (A-B) Size distribution of EVs, EV-Virus, and Virus samples was determined by using a Nano Tracking Analysis (NTA) instrument. (C) Immunoblot analysis of exosome markers TSG101, CD63 and CD9 in the different EV-preparations. (D) The surface charge of the EVs-LL/2, EVs-MC-38, Virus, EV-Virus-LL/2 and EV-Virus-MC-38 was measured using a ZetaSizer Nano Malvern instrument. Bars represent the means +/- SD of three experiments. (E) Cryo-EM image of EVs and EV-Virus (scale bar 100 nm) acquired with a FEI Talos Arctica 200 kV FEG electron microscope equipped with a FEI Falcon 3EC direct electron detector and Volta Phase-plate

We previously demonstrated that murine lung cancer cell-derived EVs could be a useful carriers for the systemic delivery of OVs to the neoplastic tissue and that EVs are able to induce immunogenic cell death *in vitro* and a marked anti-cancer effect *in vivo* 24 through a mechanism that involves the stimulation of a localized tumor-associated inflammatory response 24. In these previous studies, tumour-derived EVs were used to deliver OVs* in vitro* and *in vivo* to the same tumour that originated the vesicles; in the current work, we aimed to test the possibility of targeting different tumour types. To this end, we used a mouse lung cancer (LL/2) and colon cancer (MC-38) cells to produce EV formulations and to generate tumour models in immunocompetent syngeneic mice (C57Bl/6). EV formulations were characterized respect to the particle size distribution by using NTA (Figure 1A-B), and all formulations were within the range of 50 to 400 nm (Figure 1A). The free viruses in the EV-Virus formulations were inactivated with NaOH-treatment, a procedure that we previously shown to preserve the EV integrity and the activity of the encased virus 24. Further EV characterization was carried out by western blot analysis demonstrating the presence of specific EV biomarkers such as TGS101, CD63 and CD9 in the preparation (Figure 1C). Moreover, dynamic light scattering analysis revealed that EVs and EV-Virus had a negative zeta-potential of approximately -40 mV, while the free virus had a zeta-potential of -20 mV (Figure 1D), which is in concordance with our previous results 24. Finally, cryo-EM experiments directly demonstrated the EV integrity and the incorporation of the virus within the vesicles (Figure 1E).

**Figure 2:**
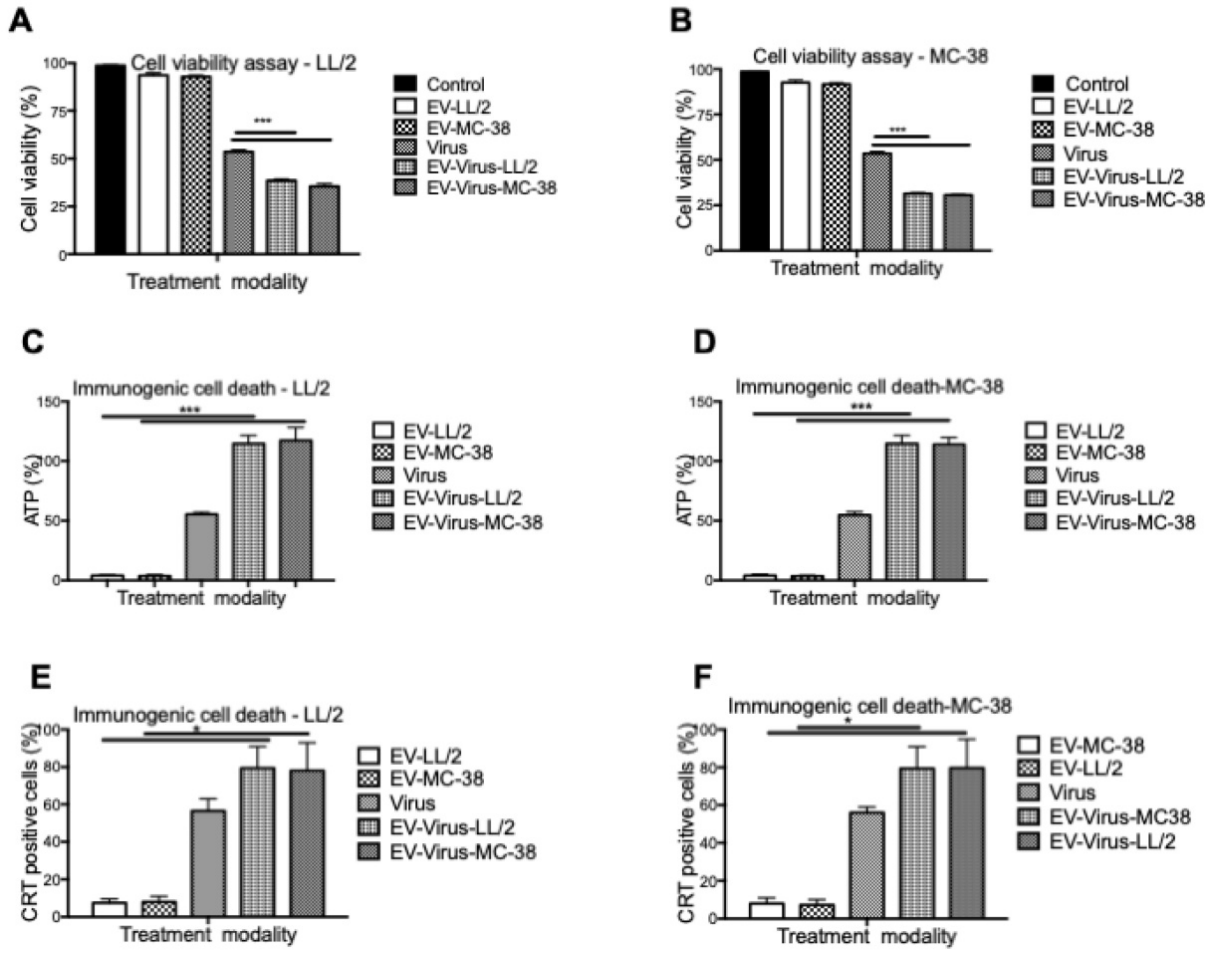
***In vitro* anti-tumour properties of EVs as drug delivery vehicles.** (A) Antineoplastic efficacy was measured by MTS cell viability assay in the LL/2 and MC-38 cell lines. Cell viability has been expressed as the percentage of the viable cells, normalized on the average measurement of the control group (set as the reference 100%). Measurements have been performed 72 h post-treatment. (C-D) Extracellular ATP was measured from the indicated cell line supernatants, 48h post-treatment using ATP determination kit. (E-F) Calreticulin exposure on outer cell surface of the murine cancer cells was measured 24h post-treatment by flow cytometer. Bars represent the means +/- SD of three experiments; *p< 0.05, ***p< 0.001.

These experiments confirmed that the encapsulation of OVs had only minimal effects on size and charge of the murine EVs, similar to what was observed with formulations previously obtained with human and murine lung cancer EVs 24,42. To evaluate the anti-tumour activity of the LL/2 and MC-38 derived EV formulations, we tested their cytotoxic effects with the MTS cell viability assay with the EVs of the same origin and by crossing the treatments. In particular, the EV-formulations obtained from LL/2 cells (EVs-LL/2, EV-Virus-LL/2) were used to treat MC-38 cells, while the EV-formulations obtained from MC-38 cells (EVs-MC-38, EV-Virus-MC-38) were used to treat LL/2 cells. Surprisingly, the EV-Virus formulation showed the highest anti-cancer activity irrespective of the cell lines that were generating the EVs, as compared to cells treated with the Virus alone (Figure 2 A-B) (p<0,001), suggesting that the cell cytotoxicity of the EV formulations was not cell line dependent.

The success of the cancer treatment relies on the induction of immunogenic tumor cell death (ICD) and induction of anti-tumor immune responses. Therefore to evaluate whether the EV-formulations could induce an immunogenic cell-death program, the expression of specific markers, such as exposure of calreticulin on the cell surface and extracellular release of ATP 46, was measured on murine lung and colon cancer cells respectively treated with 10 particles/cell EV-LL/2 and EV-MC-38, Virus, EV-Virus-LL/2, EV-Virus-MC-38. Again, the highest immunogenic cell death on tumour cells was observed with EV-Virus-LL/2 and EV-Virus-MC-38 treatments irrespectively from the cell line treated (Figure 2C-D-E-F), while the EVs administered alone did not influence immunogenic cell death. In this study, we demonstrated that Virus containing formulations were able to induce ICD to the greatest extent in comparison to EV alone. A trend to increase of the immunogenic cell death marker expression (Fig 2C-D-E-F), although statistically non-significant, was constantly observed also for the EV-Virus treatments when compared to the treatments with virus alone. This slight increase could be ascribed to the ability of EVs to concentrate the virus at the tumor site due to the EV-dependent tumor tropism^23^. These data indicated that all EV formulations, independent of their origin, were able to induce an immunogenic cell death program, thereby suggesting that the tumor uptake of the vesicles depended on a general mechanism shared by all cancer types.

### Homologous tumour-tropism of murine cancer-derived EVs

**Figure 3:**
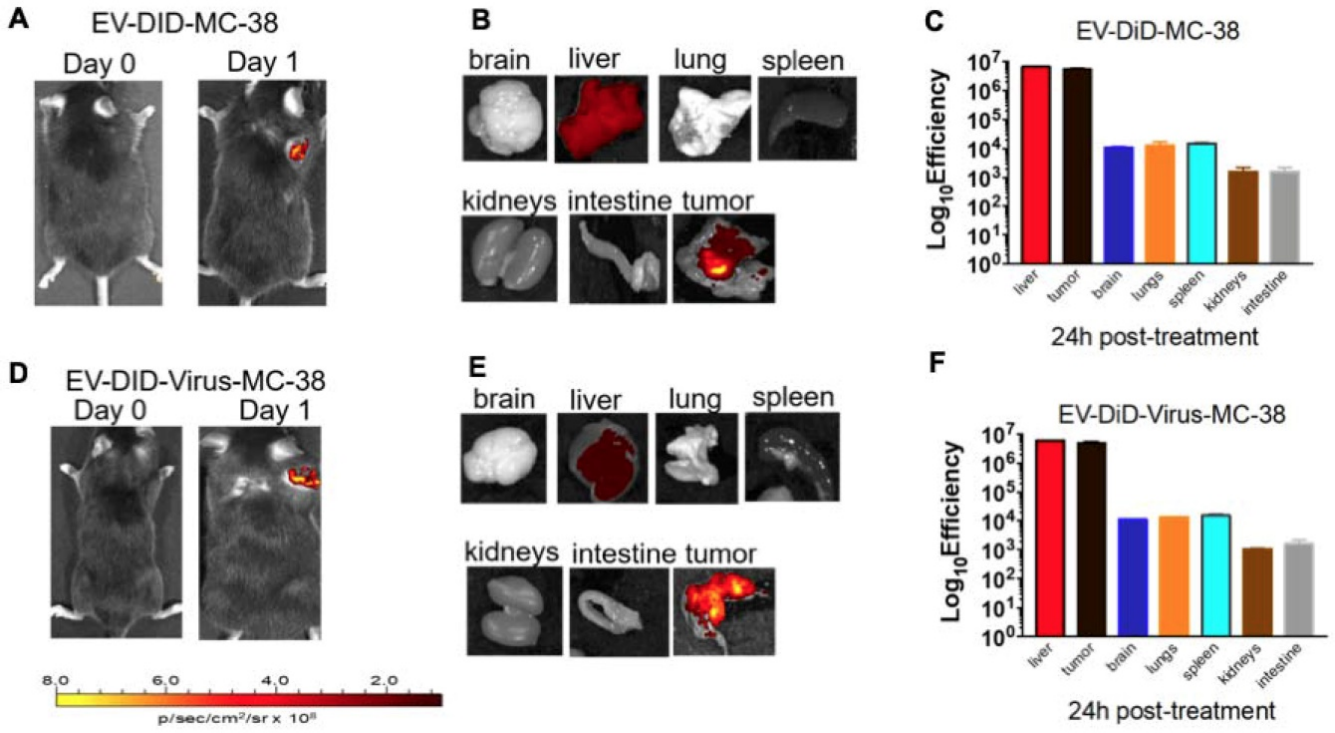
** Murine colon cancer derived EVs formulations (+/- OVs) show a positive fluorescent signal at the tumour site.** (A) Representative images of the photon emission (fluorescence) in the tumor area of C57BL/6 previously s.c. injected with 1^.^ 10^6^ MC-38 cells and i.v treated with EV-DiD-MC-38. (B) Representative images of the photon emission in 7 organs explanted from mice treated with EV-DiD-Virus-MC-38. (C) Quantification of fluorescence emission from explanted organs assessed using the Living Image Software (PerkinElmer) and CCD-camera (IVIS Lumina II Quantitative Fluorescent and Bioluminescent Imaging; PerkinElmer, Waltham, MA, USA). The results represent mean +/- SD. (D) Representative images of the photon emission (fluorescence) in the tumour area of C57BL/6 previously s.c. injected with 1^.^ 10^6^ MC-38 cells and i.v treated with EV-DiD-Virus-MC-38. (E) Representative images of the photon emission in 7 organs explanted from mice treated with EV-DiD-Virus-MC-38. (F) Quantification of fluorescence emission from explanted organs assessed as described in (C).

Previous data showed a marked homologous tumour tropism of EV formulations, when given i.v. to mice bearing a lung tumour derived from the same cell line 24,42. To demonstrate that also our preparation of colon cancer EVs had the same tumor tropism reported in our previous works on lung cancer, we have encapsulated the fluorescent dye DiIC18(5); 1,1′-dioctadecyl-3,3,3′,3′-tetramethylindodicarbocyanine, 4-chlorobenzenesulfonate salt (DiD) to the EVs and obtained the EV-DiD-LL/2 and EV-DiD-Virus-MC-38 formulations 24,47; then, we evaluated the fluorescence biodistribution after i.v. administration of 1x10^8^ particles/tumour to the C57Bl/6 wild type mice engrafted with the corresponding cell line originating the EVs. The *in vivo* imaging acquisitions of fluorescence were carried out 24h post-treatment (Figure 3A-D and Figure S1A) and acquisitions showed a specific signal arising from the tumour, suggesting that the probe was selectively accumulated in the neoplastic tissue when animals were treated with both EVs and EV-Virus particles. This effect was then confirmed by *ex vivo* imaging analysis of the fluorescence emitted by the dissected organs that showed a positive signal originating mostly from the tumour and the liver (Figure 3 B-C-E-F and Figure S1B); when administered i.v. the dye alone (not formulated into EVs) did not produce a preferential accumulation of the fluorescence in any tissue including the tumor, with the exception of liver, where the fluorescent signal could be attributed to autofluorescence since a similar fluorescent emission could also be detected in animals that were not injected with the probe (Figure S2) 24,42.

### Heterologous tumour-tropism of murine cancer-derived EVs

**Figure 4:**
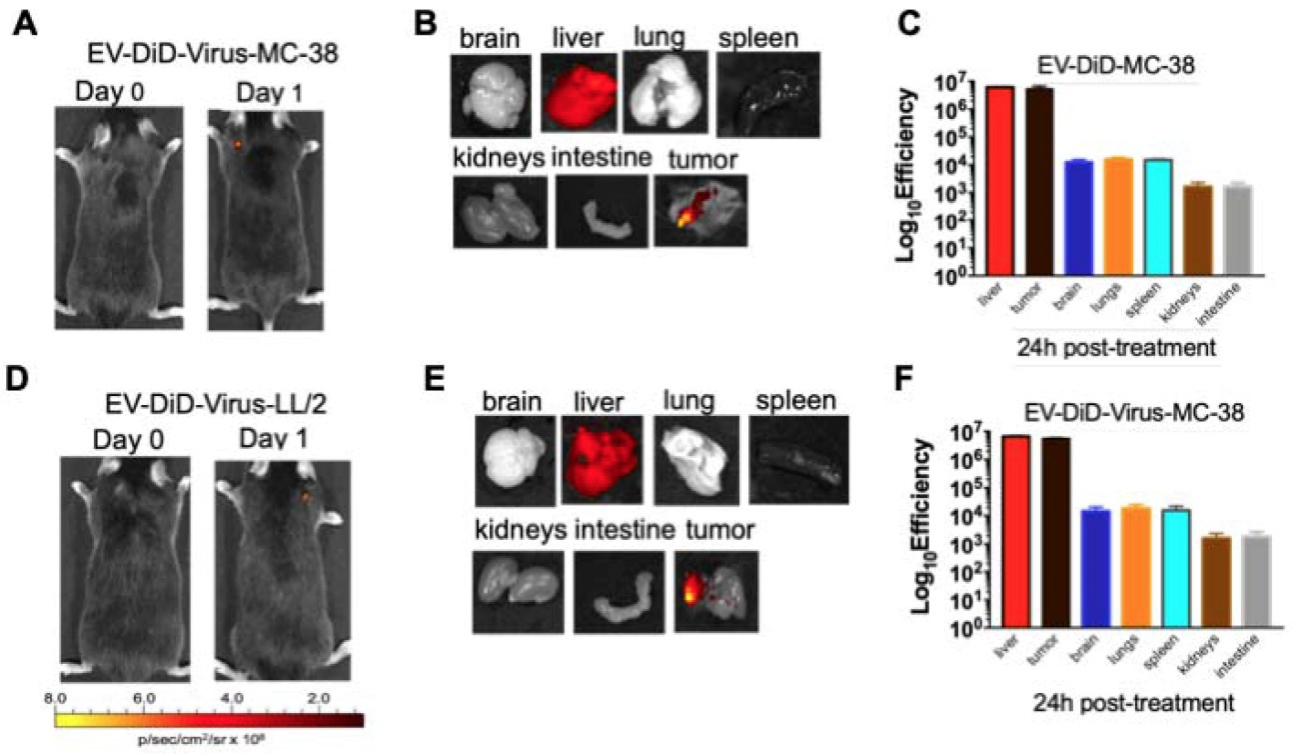
** Colon and lung cancer derived EV-formulations are able to target lung tumour sites.** (A) Representative images of the photon emission (fluorescence) in the tumour area and in 7 organs explanted from murine lung cancer (LL/2) bearing mice i.v. treated with EV-DiD-Virus-MC-38. (B) Representative images of the fluorescence emitted by 7 organs explanted from mice i.v. treated with EV-DiD-Virus-MC-38. (C) Quantification of fluorescence emission from explanted organs assessed using the Living Image Software (PerkinElmer) and CCD-camera (IVIS Lumina II Quantitative Fluorescent and Bioluminescent Imaging; PerkinElmer, Waltham, MA, USA). The results represent the mean +/- SD. (D) Representative images of the photon emission (fluorescence) in the tumour area and (E) in 7 organs explanted from murine colon cancer (MC-38) bearing mice i.v. treated with EV-DiD-Virus-LL/2. (F) Quantification of fluorescence emission from explanted organs assessed using the Living Image Software (PerkinElmer) and CCD-camera (IVIS Lumina II Quantitative Fluorescent and Bioluminescent Imaging; PerkinElmer, Waltham, MA, USA). The results represent the mean +/- SD.

The ability of EVs to specifically target the tumour site prompted us to verify whether their biodistribution was dependent from the tumor originating the vesicles. To this end, C57BL/6 mice bearing lung cancer (originating from LL/2 cell line) were i.v treated with colon cancer (MC-38)-derived EV-formulations (EV-DiD-Virus-MC-38) (Figure 4A-C); conversely, another set of mice bearing colon cancer (originating from MC-38 cell line) were i.v treated with lung cancer (LL-2) derived EV-formulations (EV-DiD-Virus-LL/2) (Figure 4D-F). The *in vivo* imaging acquisitions of fluorescence were carried out 24h post-treatment (Figure 4A-D). Interestingly, imaging showed a fluorescent accumulation in the tumour (Figure 4A-D), in addition to the autofluorescence of the liver (Figure 4 B-C-E-F). Taken together these results were indicative of a heterologous tropism of the EVs selectively recognizing the neoplastic tissue.

In agreement with this conclusion, the replication of the virus measured by qPCR showed the highest concentration of adenoviral DNA copies in the tumour of mice treated with EV-Virus formulations, suggesting that the virus was replicating locally (approx. 40,000 DNA copies detected, p<0.001); while no virus was detectable in liver and serum of the same animals (Figure S3A-B). Furthermore, since the adenoviral vector has been armed with human CD40 ligand (CD40L), we have performed quantification measurements of this exogenous protein specifically produced by the virus: the analysis provided consistent results compared with the qPCR data: the highest concentration of the CD40L was found in tumour tissues (Figure S3C-D) 5-fold higher compared to the one found in serum and liver. The basal expression of CD40L in liver and serum is likely due to a cross-talk of the antibody used for the ELISA with the endogenous mouse CD40L protein; nevertheless, the enrichment of CD40L protein in the tumor was indicative of the additional expression of this protein due to the virus itself.

Taken together these results confirmed the targeted delivery and tumor specific replication of the virus encased within EVs.

### Murine cancer derived EV-formulations also recognize tumours spontaneously arising from the tissue of a genetically modified mouse (MMTV-NeuT)

**Figure 5:**
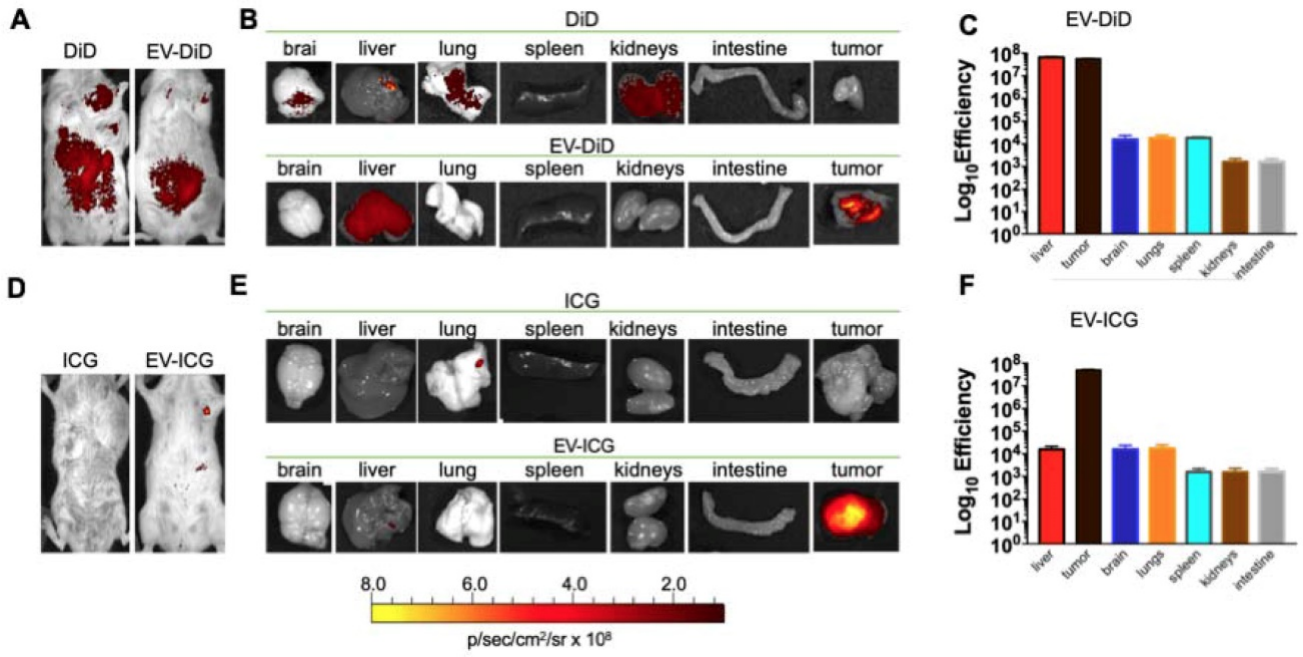
** Cancer derived EV-formulations loaded with different fluorescent markers are able to target the mammary tumors in MMTV-NeuT mice.** (A-B) Representative images of the photon emission (fluorescence) in the tumour area and in 7 organs explanted from the MMTV-NeuT mice i.v. treated with EV-DiD-LL/2 versus DiD. (C) Quantification of fluorescence emission from explanted organs assessed using the Living Image Software (PerkinElmer) and CCD-camera (IVIS Lumina II Quantitative Fluorescent and Bioluminescent Imaging; PerkinElmer, Waltham, MA, USA). (D-E) Representative images of the photon emission (fluorescence) in the tumour area and in 7 organs explanted from MMTV-NeuT mice i.v. treated with EV-ICG-Virus-LL/2 versus ICG. (F) Quantification of fluorescence emission from explanted organs assessed using the Living Image Software (PerkinElmer) and CCD-camera (IVIS Lumina II Quantitative Fluorescent and Bioluminescent Imaging; PerkinElmer, Waltham, MA, USA).

In previous experiments, we have tested the cancer-specific heterologous tropism of EVs in tumours originated by syngeneic engraftment. These tumours may differ from those spontaneously originating in the tissue in terms of encapsulation or neo-vascularization 48: these differences could have affected the permeability of the fluorescent dye to the tumour. Thus, we tested the ability of EVs to target the tumor site in a transgenic mouse model (MMTV-NeuT mice), which displayed palpable invasive carcinoma in their mammary glands (Figure 5 A-B). Our results showed that the EV-DiD-LL/2 formulation was able to accumulate mainly in the mammary tumour, when compared to the free DiD, which showed only the liver autofluorescence 24,42 (Figure 5 A-B-C). Due to the autofluorescence problems of DiD detection in liver, to minimize the background we decided to use the near infrared fluorescent (NIR) indocyanine green (ICG) for the characterization of the whole-body biodistribution of EV formulations. The results displayed a tumour-specific tropism when the fluorescent probe was loaded into the vesicles that we could not detect when ICG was administered alone (Figure 5D-E-F). Thus, we concluded that ICG could be used for the evaluation of EV biodistribution and was giving a significantly less background when compared to DiD.

**Figure 6:**
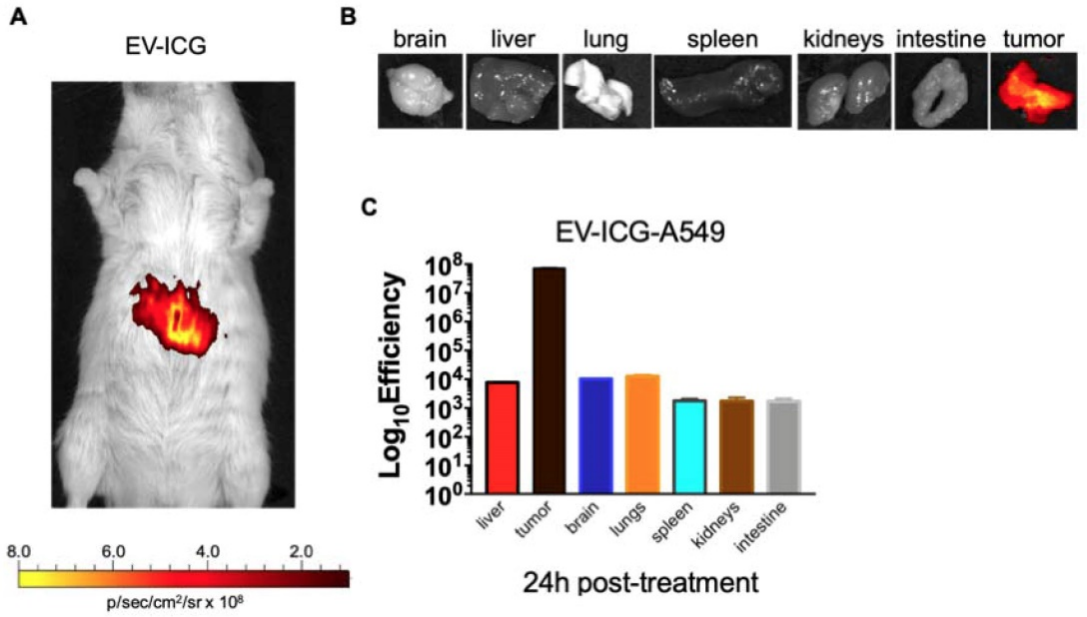
** Human lung cancer derived EVs are able to target the mammary tumors in MMTV-NeuT mice.** (A) Representative image of the photon emission (fluorescence) in the tumour area of MMTV-NeuT mice i.v treated with EV-ICG-A549. (B) Representative image of the photon emission (fluorescence) in 7 organs explanted from genetic breast cancer mice. (C) Quantification of fluorescence emission from liver and tumour tissue assessed using the Living Image Software (PerkinElmer) and CCD-camera (IVIS Lumina II Quantitative Fluorescent and Bioluminescent Imaging; PerkinElmer, Waltham, MA, USA).

Interestingly, the biodistribution profile of EVs did not depend on the tumour engraftment but showed a heterologous tumour-tropism also in spontaneously occurring tumours in the mammary tissue. Finally, we tested whether EVs derived from a human cancer cell line had cross-species homing capability: to this end, we loaded the ICG dye in human lung cancer derived EVs (EV-ICG-A549) and measured their biodistribution 24 h after treatment in MMTV-NeuT mice bearing spontaneous mammary cancer. The results demonstrated that the human EVs were also able to target the mouse mammary tumor (Figure 6A-B-C). The generalized tumor tropism of EVs could be attributed to a peculiar phagocytic or other activities present in the transformed cells 9,14,49; to rule out this hypothesis we have loaded ICG into EVs isolated from a human biopsy of a healthy liver tissue. When injected into C57BL/6 mice these non-cancerous derived EVs were unable to accumulate at the cancer site (Figure S4-S5). Moreover, also EVs isolated from non-transformed human myoblasts C2C12 did not show any accumulation at the neoplastic tissue (Figure S6), again suggesting that the tumour recognition was not a feature depending only on a peculiar cancer activity (e.g. augmented phagocytosis of cancer cells), but required the specific generation of the EVs in cancer cells as well as specific features present in neoplastic tissues.

## Discussion

The heterologous and cross-species homing of tumour-derived EVs might reflect their role in the intercellular communication contributing to the transformation mechanism of the normal tissue 50. Indeed, it has been demonstrated that tumour-derived EVs can promote the intercellular cross-talk of cancer-related molecules contributing to the acquisition of the different hallmarks of cancer 7,8. However, the role of the EV interactions among the heterogeneous cell types characterizing the tumour tissues has not been thoroughly elucidated to date 51. It has been reported that EVs originated from some immune-cells such as NK or T-cells ^30^ and from mesenchymal stem cells can bear receptors able to reiterate the tumour tropism of their parental cells ^29,46^. In this case, it is suggested that EVs from different cell sources may potentially have different innate homing capabilities *in vivo* by adopting the homing pattern of the parental cell of origin, through the acquisition of the same repertoire of surface receptors and extracellular matrix-binding proteins present in their parental cell 33. Thus, the homing capability of the vesicles may reflect their functional properties that differ considering EVs generated by the tumour micro-environment as compared to those originating from non-malignant cells. These differences have been extensively described and include distinct biogenesis mechanisms 16,17, cargo uptake 18,19 and the expression of specific membrane proteins and antigens 20,21. It is therefore conceivable that these peculiar characteristics of cancer-derived EVs may direct their tropism; thus, EVs originating from different tumours are expected to have different membrane proteins and antigens on their surface supporting the hypothesis of a strict tissue specific tropism 20,21. Conversely, our data indicate that this hypothesis is not supported and suggest a conservation of the homing features independent of the tumour type or even the species originating the vesicles. This finding might reflect the existence of a biological mechanism that allows heterogeneous cells composing the tumour to communicate with one another through the release of EVs 53, overcoming natural barriers that are physiologically restricting the communications through EVs.

A recent work from Hoshino and collaborators reported that cancer-derived EVs are able to set the niche in distal organs to receive metastatic cells; this ability is due to the presence of a specific repertoire of integrins on their surface that provide the needed tissue-specific homing abilities for reaching the organ site of metastasis and delivering the vesicles content 15. In our experiments, we have not observed this key mechanism underlying cancer spreading, likely because of the large number of EVs used in the systemic delivery we adopted; this, together with the sensitivity limitation of our method of detection, could have limited our *ex vivo* measures only to the large dye accumulation we observed in tumour tissues; in this condition, we likely failed to detect small signals coming from the niches present in normal tissues recognized as metastatic sites and described by the study of Hoshino and collaborator.

In our case the cancer-derived EV homing to neoplastic tissue could be either due to a ligand/receptor interaction or to other unspecific mechanisms: for instance the tumour uptake of the vesicles may occur because of the increased phagocytic activity of the recipient cells 14. Our experiments using EVs generated from a healthy tissue have ruled out the latter possibility: indeed, in this case, we would have expected a general uptake also of these “normal” vesicles by the neoplastic tissues that we have not observed (Figure S4). Therefore, the specific homing of our cancer-derived EVs may be due to a specific ligand-receptor interaction similar to that previously observed for other EV homing abilities 15,49. In this case, we might hypothesize the presence of a tumour-specific “ligand” exposed on the EV surface during their biogenesis in the cancer cell and a tumour-specific “receptor” already sitting on the surface of different types of cancer cells.

The identification of the mechanisms underlying the specific behaviour of cancer-derived-EVs is currently one of the major challenges facing in the field of EV biology 54 and the cancer tropism of EVs is an important issue to be considered not only for a better understanding of the role of EVs in tumour cell-to-cell communication, but also for the use of EVs as a tool for the tumour-specific delivery of theranostic agents 23,24,42. At the moment the tropism and internalization mechanisms of tumor-derived EVs can only be speculated: taken together, our results suggest the existence of a specific “general” tumor antigen expressed across species that is recognized by a “general” ligand present on the EVs surface. If this will be proven true, the characterization of such a ligand-receptor interaction will provide clues also for understanding the mechanism responsible of the internalization of the dye in tumour cells.

Our work, by highlighting the possible existence of a selective ligand-receptor mechanism responsible for the tumour-tropism, paves the way for future research aimed at constructing biocompatible nanovesicles with a cancer-selective homing. We demonstrated that EVs may be loaded with therapeutics such as OVs and diagnostic agents already approved by regulatory agency for their use in humans, such as ICG; this proof-of-principle experiment can be instrumental for future theranostics applications, for example in intraoperative imaging 55, where the diagnostic delivery of ICG may be combined with the loading of drugs (e.g. paclitaxel 56 or doxorubicin 57) or radiotherapeutic isotopes (e.g. ^99m^Tc 58) for curing tumour micrometastasis and residual neoplastic cells eventually remaining in the normal tissue after surgery.

## Conclusions

In this study, we demonstrated the existence of a heterologous, cross-species mechanism underlying the tumour-specific recognition of cancer-derived EVs regardless the tissue of origin. Our study paves the way for the description of a novel EV role in intercellular communication and to exploit the use of EVs in theranostics applications. Clearly, the molecular characterization of this mechanism in future studies will provide a rational basis for the construction of synthetic biocompatible nanoparticles avoiding the potential harmful effects associated with the use of tumour-derived particles.

## Supplementary Material

Supplementary figures.
