# Supplementary material for: Heterologous and cross-species tropism of cancer-derived extracellular vesicles
Source: Theranostics. 2019 Aug 9;9(19):5681–93. doi: 10.7150/thno.34824 (PMC6735396; doi:10.7150/thno.34824)
Supplement: Supplementary file 1 — Supplementary figures. [file thnov09p5681s1.pdf]

Title: Heterologous and cross-species tropism of cancer-derived extracellular vesicles

Mariangela Garofalo[1*], Alessandro Villa[1], Daniela Crescenti[1,2], Monica Marzagalli [2], Lukasz Kuryk [3,4] , Patrizia Limonta[2] , Vincenzo Mazzaferro[1,5], Paolo Ciana[1*]

SUPPLEMENTARY FIGURES

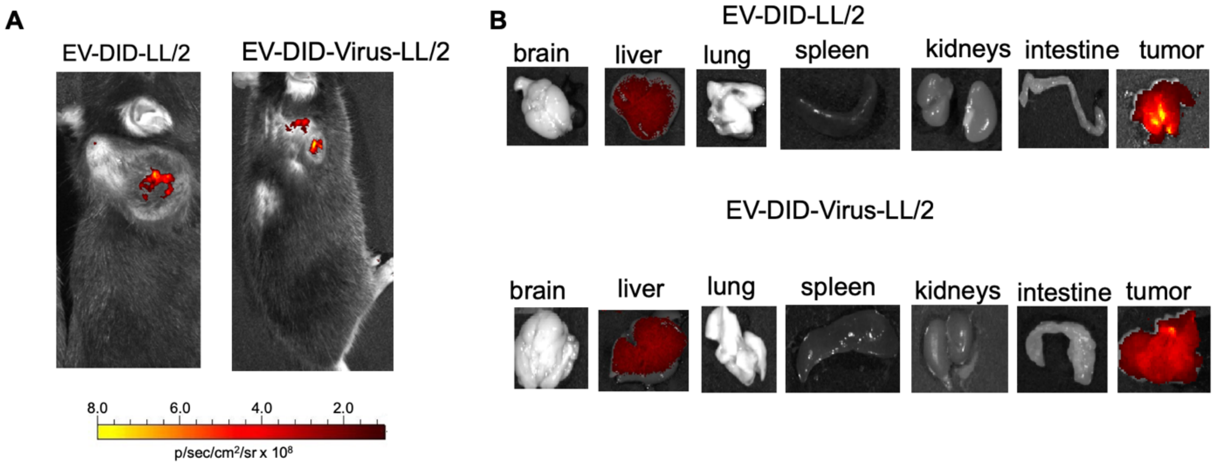

Figure S1. EVs formulations show a positive fluorescent signal at the tumour site. (A) Representative images of the photon emission (fluorescence) in the tumour area of tumour-bearing C57BL6 mice that were intravenously injected with EV-DiD-LL/2 ($1 \cdot 10^8$ particles/tumour) and EV-DiD-Virus-LL/2 ($1 \cdot 10^8$ particles/tumor + $1 \cdot 10^8$ vp/ tumour). (B) Representative images that indicate the intensity of photon emission in 7 organs explanted from treated mice.

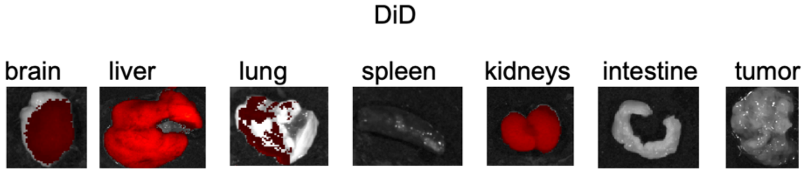

Figure S2. DiD alone does not show positive fluorescent signal at the tumour site. Representative images that indicate the intensity of photon emission in 7 organs explanted from C57BL/6 previously s.c. injected with $1 \cdot 10^6$ MC-38 and i.v. treated with DiD.

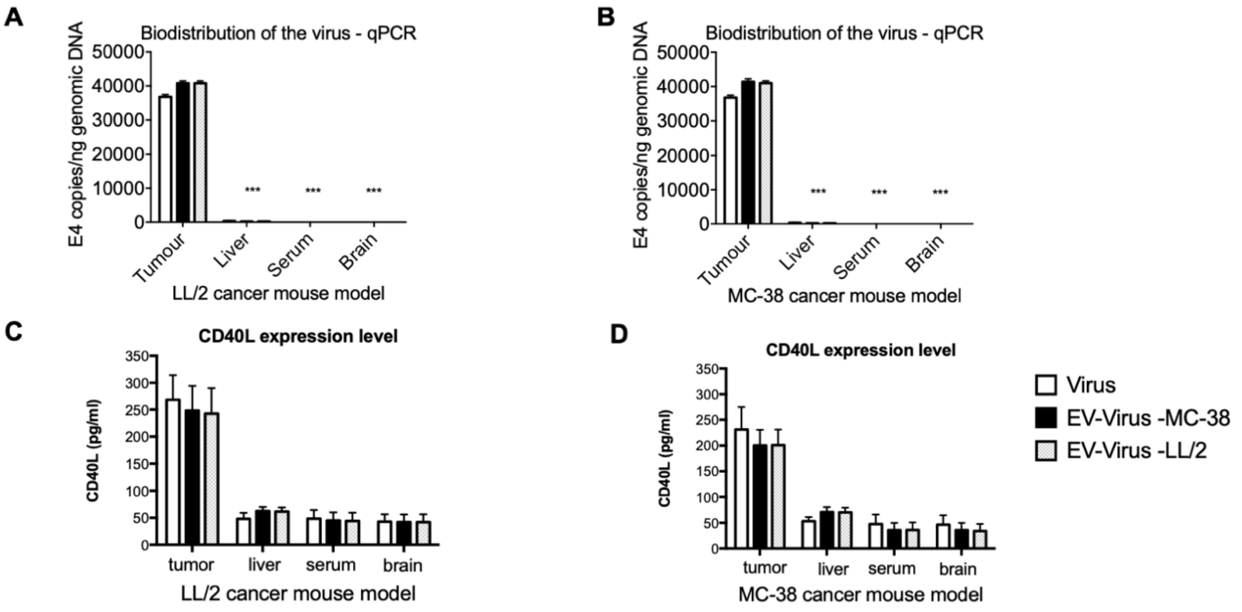

Figure S3. Biodistribution of virus and measurement for CD40L. (A-B) qPCR of the adenoviral copy number normalized towards the E4 gene measured from the organs (tumour, liver, serum and brain) of euthanized mice at the end of the treatment. Error bars indicate +/- SD *p<0.05, **p<0.01, ***p<0.001. (C-D) Quantitative analysis of human CD40L produced by the investigated oncolytic adenovirus using an ELISA kit from harvested organs (tumour, liver, serum). The results represent the mean +/- SD.

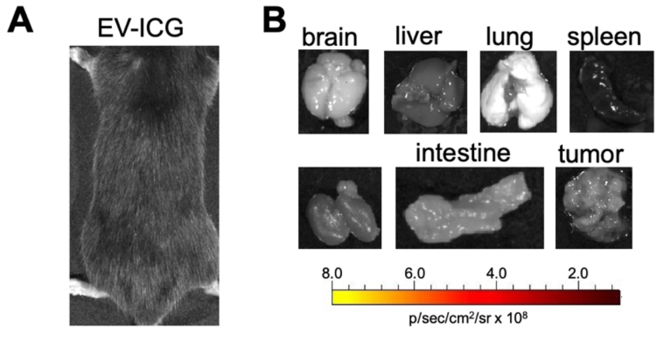

Figure S4. Non-cancerous-derived EVs cannot target the tumour. (A) Representative image of the photon emission (fluorescence) in the tumour area of C57BL/6 mice i.v murine lung cancer (LL/2) bearing mice i.v. treated with EV-ICG isolated from the patient's healthy liver. (B) Representative image of the photon emission (fluorescence) in 7 organs explanted from treated mice.

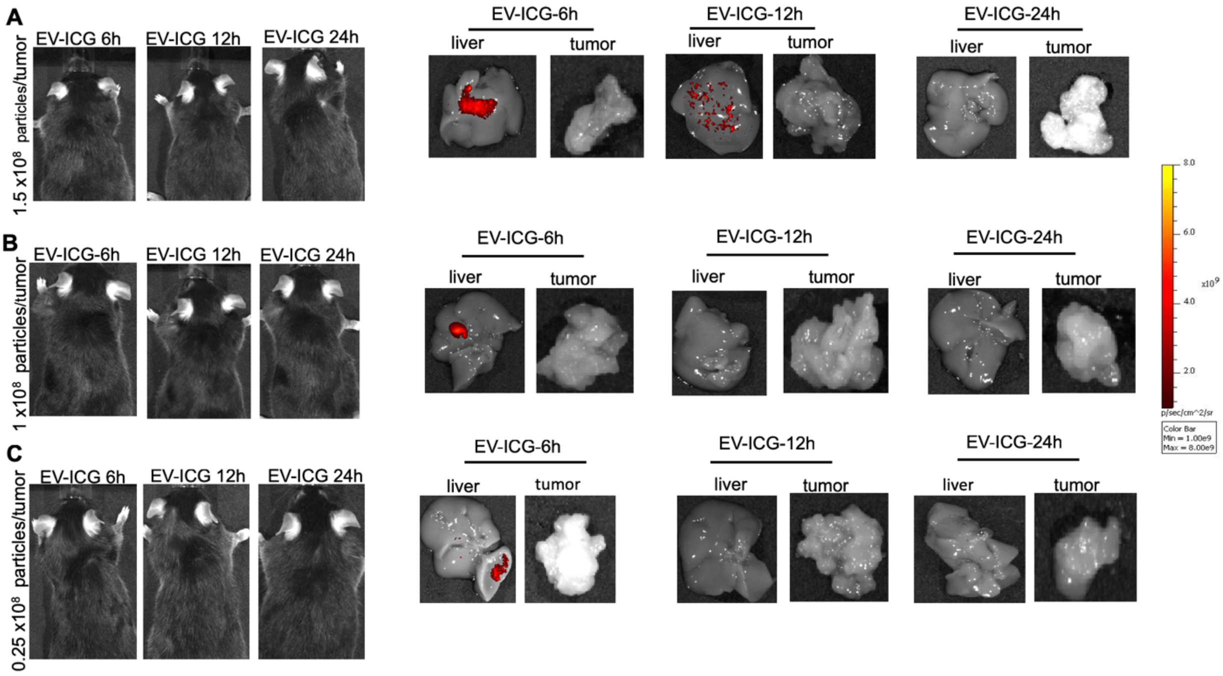

Figure S5. Non-cancerous derived- EV formulations, tested in different conditions did not show homing abilities both *in vivo* and *ex vivo*. (A-B-C) Representative images that indicate the intensity of photon emission (fluorescence) measured after 6, 12, 24 h post-treatment in mice intravenously treated with non-cancerous derived EVs loaded with ICG (EV-ICG) ($1{\cdot}10^8$ particles/tumor + 10 ug/mL).

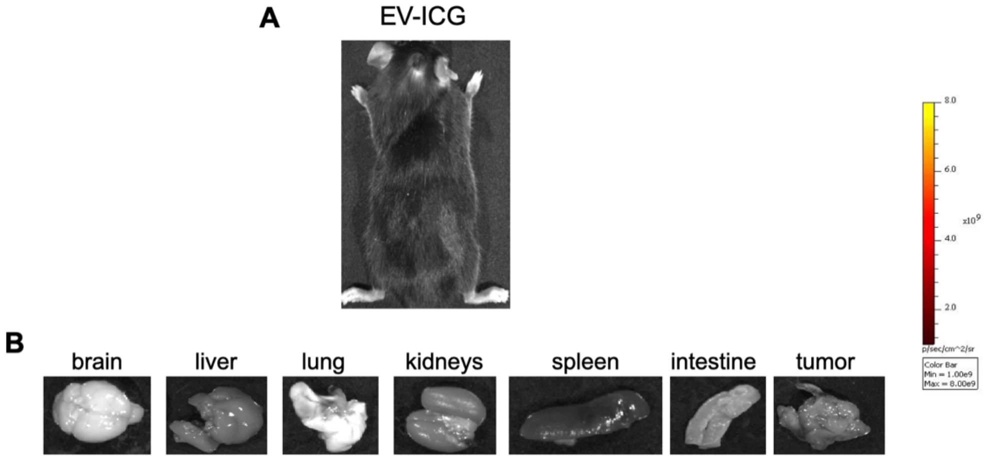

Figure S6. EV-formulations isolated from C2C12 cells did not show positive fluorescent signal at the tumour site**.** (A-B) Representative images that indicate the intensity of photon emission (fluorescence) measured after 24h post-treatment in tumor area of mice intravenously treated with non-cancerous derived EVs loaded with ICG (EV-ICG) ($1{\cdot}10^8$ particles/tumor + 10 ug/mL).
